# Central nervous system gene expression changes in a transgenic mouse model for bovine spongiform encephalopathy

**DOI:** 10.1186/1297-9716-42-109

**Published:** 2011-10-28

**Authors:** Raül Tortosa, Xavier Castells, Enric Vidal, Carme Costa, María del Carmen Ruiz de Villa, Àlex Sánchez, Anna Barceló, Juan María Torres, Martí Pumarola, Joaquín Ariño

**Affiliations:** 1 Departament de Medicina i Cirurgia Animals, Universitat Autònoma de Barcelona, 08193, Cerdanyola del Vallès, Barcelona, Spain; 2 Institut de Biotecnologia i Biomedicina i Departament de Bioquímica i Biologia Molecular, Universitat Autònoma de Barcelona, Bellaterra 08193, Barcelona, Spain; 3 Priocat Laboratory, Centre de Recerca en Sanitat Animal, UAB-IRTA, Campus de la Universitat Autònoma de Barcelona, 08193, Barcelona, Spain; 4 Departament d'Estadística, Facultat de Biologia, Universitat de Barcelona, 08028 Barcelona, Spain; 5 Centro de Investigación en Sanidad Animal, INIA, Valdeolmos, 28130 Madrid, Spain; 6 Centre de Biotecnologia Animal i de Teràpia Gènica (CBATEG), Universitat Autònoma de Barcelona, Bellaterra 08193, Barcelona, Spain

## Abstract

Gene expression analysis has proven to be a very useful tool to gain knowledge of the factors involved in the pathogenesis of diseases, particularly in the initial or preclinical stages. With the aim of finding new data on the events occurring in the Central Nervous System in animals affected with Bovine Spongiform Encephalopathy, a comprehensive genome wide gene expression study was conducted at different time points of the disease on mice genetically modified to model the bovine species brain in terms of cellular prion protein. An accurate analysis of the information generated by microarray technique was the key point to assess the biological relevance of the data obtained in terms of Transmissible Spongiform Encephalopathy pathogenesis. Validation of the microarray technique was achieved by RT-PCR confirming the RNA change and immunohistochemistry techniques that verified that expression changes were translated into variable levels of protein for selected genes. Our study reveals changes in the expression of genes, some of them not previously associated with prion diseases, at early stages of the disease previous to the detection of the pathological prion protein, that might have a role in neuronal degeneration and several transcriptional changes showing an important imbalance in the Central Nervous System homeostasis in advanced stages of the disease. Genes whose expression is altered at early stages of the disease should be considered as possible therapeutic targets and potential disease markers in preclinical diagnostic tool development. Genes non-previously related to prion diseases should be taken into consideration for further investigations.

## Introduction

Transmissible Spongiform Encephalopathies (TSE) are a group of neurodegenerative diseases characterized by a long incubation period followed by a fatal outcome [1]. Bovine Spongiform Encephalopathy (BSE), a disease first reported by Gerald Wells in 1987 [2] is one of those TSE affecting animals with an important social and economic impact. BSE is closely related to the variant of Creutzfeldt-Jakob disease that affects humans [3]. The prevalent hypothesis claims an abnormal isoform of the cellular prion protein (PrPc) as the only etiological agent [4].

The pathogeny of TSE in the nervous tissue is characterized by the accumulation of the pathological isoform of the prion protein (PrPres), glial cell activation, neurodegeneration and neuronal loss. Pathogenic mechanisms of the nervous degeneration are not completely defined even though many studies have been performed. These studies include clinical examinations, histopathological evaluation of tissues, identification of the pathological prion protein by western blot and immunohistochemical techniques [5-7]. In recent years gene expression analysis has been applied to this group of diseases using DNA array techniques [8-15] with the aim of identifying groups of genes related to the TSE pathogenesis.

The main objective of this study was to improve the knowledge on the pathogenic mechanisms of BSE using gene expression analysis. A transgenic murine model of BSE was used for the study. This model has been characterized in previous studies [16,17] and its distinctive feature is the overexpression of the bovine PrPc (8 times more PrPc than that expressed in cattle) instead of the murine protein. This results in a greater susceptibility to develop BSE upon intracerebral inoculation in comparison with wild type mice, i.e. a reduced incubation period (287 ± 12 days for homozygous animals/311 ± 17 days for heterozygous animals) [16].

Several studies have been published on gene expression analysis concerning scrapie [12,18,19] but this kind of information about BSE has only been recently available [10,13-15,20]. In this paper a dynamical study of the evolution of the disease was performed by an oligonucleotide microarray genome wide gene expression analysis done on a well characterized transgenic mouse model of BSE on different time points of the disease. The results were further verified by RT-PCR and immunohistochemistry techniques.

## Materials and methods

### Animals, inoculation, sacrifice and sample preparation

Transgenic mice (BoTg110 line with B6CBAflx129/Ola *background*) generated by Castilla et al. were used [16]. This model is characterized by the over expression of the bovine cellular prion protein (PrPc) instead of the murine PrPc under the regulation of the *prpn *murine promoter. A pool of BSE material (TSE/08/59, from now on BSE1), originating from the brainstem of 49 BSE infected cattle, supplied by the Veterinary Laboratories Agency (Addlestone, UK), was used for the infected group of animals. Brain homogenates (10% wt/vol) in sterile phosphate buffered saline (PBS) without Ca^2+ ^or Mg^2+ ^were prepared using a homogenizer (OMNI International, Warrenton, USA). Healthy cow brain homogenate was used for the negative control group. To minimize the risk of bacterial infection, all inocula were preheated for 10 min at 70°C before inoculation in mice. For the gene expression analysis, the animals were divided into two groups of 21 animals each (BSE inoculated mice and the control group) and were inoculated intracerebrally at 6-7 weeks of age. Inoculum was injected at the temporal lobe using a 25 gauge disposable hypodermic needle with 20 μL of 10% brain homogenate. Mice were sacrificed by cervical dislocation in accordance with the recommendations of the ethics committee on post inoculation days 1, 60, 120 (5 BSE inoculated animals + 5 controls per time point) i 270 (6 BSE inoculated animals + 6 controls). Brain removal was done rapidly under RNAse free conditions. Brains were divided into two pieces by a longitudinal axis section and frozen immediately in liquid nitrogen and stored at -80°C. One half of the brain was used to confirm the presence of PrPres by western blot techniques and one half was used for microarray gene expression analysis.

For immunohistochemical and histochemical analysis, eleven BoTg110 transgenic mice were inoculated with the same infective material following the protocol described above. Animals were sacrificed by an anaesthetic overdose (intraperitoneal administration of sodium pentobarbital) at different time points: 3 animals were culled at an earlier time of infection (150 days post inoculation (dpi)) and 8 animals were sacrificed at terminal stages (250-350 dpi). Age matched non-inoculated animals were used as controls. Brains were removed and fixed in 10% formalin solution. Coronal sections done at different levels (optic chiasm, piriform cortex and *medulla oblongata*) were dehydrated and paraffin embedded for its histopathological, immunohistochemical and histochemical analysis.

### RNA preparation

Total RNA was obtained from each hemiencephalon using the Qiagen RNeasy^© ^Midi kit and following the manufacturer's instructions. RNA concentration and absence of protein was determined by spectrophotometry (ND-1000 Spectrophotometer, Nanodrop Technologies, Wilminton, USA) and RNA integrity was analysed by capillary electrophoresis (Bioanalyzer 2100, Agilent Technologies, Santa Clara, USA).

### DNA labeling and hybridization

cRNA synthesis, fragmentation and hybridization were done for each of the samples for all time points and conditions (21 controls + 21 BSE inoculated mice) according tothe manufacturer's instructions and the labeling and washing were done using theprotocol EukGE-WS2-v5 in the Fluidics Station 450 (Affymetrix, Inc., Santa Clara, USA). The Mouse Genome 430 2.0 arrays were used along the assay. The procedure described in this section was performed at the Affymetrix core facility of the *Institut de Recerca de l'Hospital Universitari Vall d'Hebron *(Barcelona, Spain).

### Verification, normalization and statistical analysis of microarray data

The analysis was performed following the usual "pipeline" for microarray data. The quality control included visual inspection of array image, data preprocess (summarization, filtering and normalization), selection of genes differentially expressed for each set of conditions, search for gene expression patterns and grouping of samples and annotation of results and analysis of biological significance.

The quality of the data was verified by visual inspection of array images and diagnostic plots such as histograms, box plots and degradation plots, according to Microarray Analysis Suite 5.0 (Affymetrix) recommendations.

The data obtained from the Affymetrix chips were normalized in order to eliminate systematic biases using the RMA method [21]. This method performs three pre-processing steps: a) probe specific correction of the PM probes using a model based on measured intensity being the sum of signal and noise, b) normalization of corrected PM probes using quantile normalization and c) calculation of an absolute measure for each probe set using the robust method known as median polish.

In order to reduce noise a non-specific two step filtering process was performed. First, those genes whose signals did not reach a minimum threshold in all groups were discarded and, from the rest, only the genes whose standard deviation was greater than the median of all standard deviation were selected for the statistical analysis.

The goal of the analysis was to detect changes in gene expression along time and/or between healthy and infected groups. This two-factor setting (time and treatment) was analyzed with an ANOVA-like analysis that was done following the linear model methodology developed by G. Smyth [22]. In order to account for multiple testing problems, *p*-values were adjusted, following the Benjamini and Hochberg method [23].

At the different time points, we combined the ratio for those probesets that corresponded to the same gene (differentially expressed probes and genes between the BSE inoculated mice group and control mice group for each time point have been provided (see Additional file 1 and Additional file 2)). Genes whose fold-change value was higher than 1.7 or lower than 0.6 at any of the four stated time points with a *p*-value < 0.0012 were considered for further evaluation and were functionally classified using the Babelomics 3.2 tool [24] (see Table 1).

**Table 1:** Functional classification of differentially expressed genes at different time points of the disease (1, 60, 120 and 270 dpi) with a fold-change higher than 1.7 (up regulated genes) or lower than 0.6 (down regulated genes) at any of the four time points

			Fold changes and *p*-values					
Functional group	Gene symbol	Gene description	1 dpi	60 dpi	120 dpi	270 dpi	*p*-value	Previous prion gene expression studies on the Central Nervous System
Immune, inflammatory and stress response	Cxcl13	chemokine (C-X-C motif) ligand 13	1.59	0.91	0.90	30.48	< 1.00E-04	[18] (S), [37] (S), [38] (S)
	Clec7a	C-type lectin domain family 7, member a	1.00	1.02	0.90	3.56	< 1.00E-04	[18] (S), [32] (S), [37] (S)
	Cybb	cytochrome b-245, beta polypeptide	1.14	1.33	0.84	2.87	< 1.00E-04	[37] (S)
	C4	complement component 4 (within H-2S)	0.95	1.17	0.88	2.77	< 1.00E-04	[18] (S), [28] (S), [32] (S), [34] (S,CJD), [38] (S)
	Serpina3n	serine (or cysteine) peptidase inhibitor, clade A, member 3N	0.82	0.85	0.56	2.50	< 1.00E-04	[18] (S), [32] (S), [34] (S,CJD), [37] (S), [36] (S)
	Rrm2b	ribonucleotide reductase M2 B (TP53 inducible)	0.97	0.52	1.34	2.37	4.00E-04	
	C3	complement component 3	0.90	1.12	0.93	2.35	2.00E-04	[18] (S), [28] (S), [32] (S), [38] (S)
	C1qb	complement component 1, q subcomponent, beta polypeptide	1.02	0.91	0.94	2.20	< 1.00E-04	[27] (S), [18] (S), [28] (S), [19] (S), [9] (S), [12] (S), [32,34] (S/CJD)
	C1qa	complement component 1, q subcomponent, alpha polypeptide	1.04	1.01	0.98	2.14	< 1.00E-04	[18] (S), [28] (S), [19] (S), [12] (S), [32,34] (S/CJD)
	C1qg	complement component 1, q subcomponent, gamma polypeptide	1.07	0.88	0.88	2.14	< 1.00E-04	[18] (S), [32] (S)
	Osmr	oncostatin M receptor	0.82	1.08	0.97	2.13	< 1.00E-04	[18] (S), [28] (S), [32] (S), [37] (S)
	C3ar1	complement component 3a receptor 1	0.72	1.02	0.99	2.03	< 1.00E-04	[18] (S), [28] (S), [32] (S)
	Cd14	CD14 antigen	0.88	0.96	0.95	1.93	< 1.00E-04	[18] (S), [32] (S), [37] (S), [38] (S)
	Mpeg1	macrophage expressed gene 1	0.87	1.09	0.94	1.90	1.00E-04	[18] (S), [34] (S/CJD), [37] (S)
	Lilrb4	leukocyte immunoglobulin-like receptor, subfamily B, member 4	0.67	0.92	1.10	1.90	5.00E-04	
	Ly86	lymphocyte antigen 86	1.11	0.94	0.88	1.88	< 1.00E-04	[18] (S), [8] (S), [34] (S/CJD), [35] (S), [37] (S)
	Tlr2	toll-like receptor 2	0.97	1.12	0.93	1.84	< 1.00E-04	[18] (S), [32] (S), [34] (S/CJD), [37] (S)
	Hspb6	heat shock protein, alpha-crystallin-related, B6	0.98	1.03	1.10	1.82	< 1.00E-04	
	Cd48	CD48 antigen	0.82	0.89	0.88	1.81	< 1.00E-04	[18] (S)
	Icsbp1	interferon consensus sequence binding protein 1	0.91	1.02	0.95	1.76	< 1.00E-04	[18] (S), [28] (S)
	Ifi27	interferon, alpha-inducible protein 27	0.97	0.96	0.89	1.75	6.00E-04	
	Usp18	ubiquitin specific peptidase 18	0.87	1.04	0.80	1.72	3.00E-04	[37] (S)
	Socs3	suppressor of cytokine signaling 3	0.78	0.86	0.90	1.72	7.00E-04	[18] (S)
	Dusp1	dual specificity phosphatase 1	1.38	0.65	0.73	0.50	1.00E-04	[38] (S)
	Map4k2	mitogen activated protein kinase kinase kinase kinase 2	0.98	1.25	0.90	0.44	1.00E-04	
	Mamdc1 (Mdga2 VALIDATED))	MAM domain containing 1 (MAM domain containing glycosylphosphatidylinositol anchor 2 (VALIDATED))	0.44	2.21	0.40	0.25	0.001	
								
Glial response	Gfap	glial fibrillary acidic protein	0.89	1.06	0.99	3.37	< 1.00E-04	[85] (S), [86] (S/AD) [18] (S), [19] (S), [28] (S), [30] (CJD), [12] (S), [31] (S), [34] (S/CJD), [35] (S), [37] (S)
	Cst7	cystatin F (leukocystatin)	0.99	1.08	0.97	3.24	< 1.00E-04	[18] (S), [28] (S), [32] (S), [37] (S)
	Lgals3	lectin, galactose binding, soluble 3	0.57	1.10	0.78	2.65	< 1.00E-04	[18] (S), [28] (S), [37] (S)
	Cd68	CD68 antigen	0.80	0.99	0.82	2.04	< 1.00E-04	[18] (S), [28] (S), [32] (S), [37] (S), [38] (S)
	Tyrobp	TYRO protein tyrosine kinase binding protein	1.02	1.04	0.84	1.99	< 1.00E-04	[18] (S), [9] (S), [34] (S/CJD), [37] (S), [38] (S)
	Mt2	metallothionein 2	0.84	1.14	1.43	1.86	< 1.00E-04	[27] (S), [30] (CJD), [10] (BSE), [38] (S)
								
Cell death	Rrm2b	ribonucleotide reductase M2 B (TP53 inducible)	0.97	0.52	1.34	2.37	4.00E-04	
	Ctsc	cathepsin C	0.96	0.81	1.05	1.81	< 1.00E-04	[18] (S), [28] (S), [37] (S), [36] (S)
	Bcl2a1a	B-cell leukemia/lymphoma 2 related protein A1a	1.01	0.97	1.01	1.70	6.00E-04	[18] (S), [32] (S)
	1110006I15Rik (tmem109 (PROVISIONAL))	RIKEN cDNA 1110006I15 gene (transmembrane protein 109 (PROVISIONAL))	0.80	1.29	1.40	0.56	5.00E-04	
	AA536749 (mprip VALIDATED))	expressed sequence AA536749 (myosin phosphatase Rho interacting protein (VALIDATED))	0.85	1.45	1.02	0.53	3.00E-04	
	Nr4a1	nuclear receptor subfamily 4, group A, member 1	1.53	0.65	0.68	0.49	< 1.00E-04	[9] (S), [38] (S)
								
Cell adhesion	Cd44	CD44 antigen	0.87	0.92	0.82	2.13	1.00E-04	[18] (S), [38] (S)
	Itgax	integrin alpha X	0.95	1.06	0.89	1.79	< 1.00E-04	[18] (S), [37] (S)
	Gpnmb	glycoprotein (transmembrane) nmb	0.71	1.08	1.01	1.74	9.00E-04	[18] (S), [37] (S)
	Ptprd	protein tyrosine phosphatase, receptor type, D	0.93	2.67	0.75	0.39	1.00E-04	
								
Intracellular transport	Snx6	sorting nexin 6	1.61	0.69	1.32	2.24	4.00E-04	
	Ndel1	nuclear distribution gene E-like homolog 1 (A. nidulans)	1.06	0.56	0.96	2.11	1.00E-04	
	Snx14	sorting nexin 14	0.83	0.78	1.16	1.84	< 1.00E-04	
	Vps37c	vacuolar protein sorting 37C (yeast)	0.82	1.03	1.21	0.55	< 1.00E-04	
	Scfd1	sec1 family domain containing 1	0.62	1.51	0.67	0.52	4.00E-04	
	Rtn3	reticulon 3	0.85	1.66	0.96	0.34	< 1.00E-04	[9] (S), [15] (BSE)
								
Transmission of nerve impulse	Tyrobp	TYRO protein tyrosine kinase binding protein	1.02	1.04	0.84	1.99	< 1.00E-04	[18] (S), [9] (S), [34] (S/CJD), [37] (S), [38] (S)
	Slc6a4	solute carrier family 6 (neurotransmitter transporter, serotonin), member 4	1.12	1.62	0.99	0.60	0.0012	
	Arc	activity regulated cytoskeletal-associated protein	2.20	0.66	0.96	0.45	< 1.00E-04	
	Pmch	pro-melanin-concentrating hormone	0.98	1.27	0.83	0.44	< 1.00E-04	
	Egr2	early growth response 2	1.58	0.49	0.56	0.36	1.00E-04	
	Scn2b	sodium channel, voltage-gated, type II, beta	0.96	1.57	0.97	0.09	< 1.00E-04	
								
Signal transduction	S100a6	S100 calcium binding protein A6 (calcyclin)	0.92	0.93	0.89	1.93	< 1.00E-04	[18] (S), [29] (S)
	Hpgd	hydroxyprostaglandin dehydrogenase 15 (NAD)	0.86	0.98	0.95	1.93	< 1.00E-04	[37] (S)
	Adamts4	a disintegrin-like and metallopeptidase (reprolysin type) with thrombospondin type 1 motif, 4	0.55	1.25	0.81	0.60	4.00E-04	
	Nr4a1	nuclear receptor subfamily 4, group A, member 1	1.53	0.65	0.68	0.49	< 1.00E-04	[9] (S), [38] (S)
	Ptprd	protein tyrosine phosphatase, receptor type, D	0.93	2.67	0.75	0.39	1.00E-04	
								
Transcription	Npas3	neuronal PAS domain protein 3	1.80	0.57	1.64	2.42	4.00E-04	
	Tle4	transducin-like enhancer of split 4, homolog of Drosophila E(spl)	0.97	0.91	0.79	1.99	< 1.00E-04	
	Cbx7	chromobox homolog 7	0.93	0.87	1.19	1.96	4.00E-04	
	Smad4	MAD homolog 4 (Drosophila)	0.67	0.73	2.02	1.33	2.00E-04	
	Klf2	Kruppel-like factor 2 (lung)	1.28	0.82	0.78	0.57	1.00E-04	
	Tle1	transducin-like enhancer of split 1, homolog of Drosophila E(spl)	0.87	1.88	0.77	0.56	0.0012	
	Junb	Jun-B oncogene	1.41	0.51	0.65	0.53	< 1.00E-04	[9] (S)
	Cnot4	CCR4-NOT transcription complex, subunit 4	0.71	1.39	0.66	0.46	4.00E-04	
	Fos	FBJ osteosarcoma oncogene	1.47	0.57	0.54	0.39	1.00E-04	[10] (BSE), [38] (S)
	Mysm1	myb-like, SWIRM and MPN domains 1	1.48	2.42	0.84	0.33	< 1.00E-04	
								
Biosynthetic process	Rrm2b	ribonucleotide reductase M2 B (TP53 inducible)	0.97	0.52	1.34	2.37	4.00E-04	
	Rpl17	ribosomal protein L17	1.32	1.38	0.34	0.66	4.00E-04	
	Pbx1	pre B-cell leukemia transcription factor 1	0.46	1.51	0.79	0.54	< 1.00E-04	
	2310043N10Rik (Neat1 (PROVISIONAL))	RIKEN cDNA 2310043N10 gene (nuclear paraspeckle assembly transcript 1 (non-protein coding) (PROVISIONAL))	0.90	1.64	0.71	0.38	< 1.00E-04	
	4933439C20Rik (pisd-ps3 PROVISIONAL)	RIKEN cDNA 4933439C20 gene (phosphatidylserine decarboxylase, pseudogene 3 (PROVISIONAL)	0.79	2.67	0.99	0.14	5.00E-04	
								
Others	MGI:1929709	plasma membrane associated protein, S3-12	0.87	1.54	1.98	2.73	< 1.00E-04	[18] (S)
	1700047I17Rik (fam177a (PREDICTED))	RIKEN cDNA 1700047I17 gene (family with sequence similarity 177, member A (PREDICTED))	1.77	0.52	1.81	2.57	1.00E-04	
	Cd52	CD52 antigen	1.16	0.91	0.88	2.54	< 1.00E-04	[18] (S), [28] (S), [34] (S/CJD), [37] (S)
	4930511A21Rik (ppp2r3c (VALIDATED))	RIKEN cDNA 4930511A21 gene (protein phosphatase 2, regulatory subunit B'', gamma (VALIDATED))	1.36	0.64	1.33	1.83	7.00E-04	
	AU020206	expressed sequence AU020206	1.01	0.94	0.99	1.83	3.00E-04	[18] (S)
	A2m	alpha-2-macroglobulin	0.88	0.91	1.02	1.76	1.00E-04	[18] (S), [28] (S), [37] (S), [38] (S)
	Ifi44	interferon-induced protein 44	0.82	0.97	0.87	1.74	0.0011	
	AI467657 (Zbtb16 (PROVISIONAL)	expressed sequence AI467657 (zinc finger and BTB domain containing 16 (PROVISIONAL))	0.76	1.53	2.09	1.61	< 1.00E-04	
	Mapk4	mitogen-activated protein kinase 4	0.87	0.93	1.81	1.32	< 1.00E-04	
	Atxn7l4	ataxin 7-like 4	0.55	1.24	0.87	0.74	8.00E-04	
	Cdca1	cell division cycle associated 1	0.59	1.25	0.89	0.61	< 1.00E-04	
	1700063D05Rik	RIKEN cDNA 1700063D05 gene	1.01	0.90	0.95	0.60	< 1.00E-04	
	2700081O15Rik	RIKEN cDNA 2700081O15 gene	0.98	1.13	0.98	0.59	< 1.00E-04	
	Btg2	B-cell translocation gene 2, anti-proliferative	1.20	0.83	0.86	0.58	2.00E-04	[38] (S)
	Lrfn5	leucine rich repeat and fibronectin type III domain containing 5	0.54	1.67	0.81	0.57	5.00E-04	[33] (S)
	Gm1075	gene model 1075, (NCBI)	1.31	0.74	0.56	0.56	< 1.00E-04	
	Prp19	PRP19/PSO4 homolog (S. cerevisiae)	0.95	1.42	1.25	0.48	< 1.00E-04	[10] (BSE)
	R75368 (palm2 (VALIDATED))	expressed sequence R75368 (paralemmin 2 (VALIDATED))	1.06	2.49	0.62	0.47	5.00E-04	

Assignation of the gene function was made according to the Babelomics 3.2 tool [24] and subsequently manually curated. (S = scrapie, BSE = Bovine Spongiform Encephalopathy, CJD = Creutzfeldt-Jakob disease).

This set of genes was used as the input for an unsupervised hierarchical cluster. For this, we fixed the order of the time points (columns) and left the software to cluster genes based on different metrics to measure both the distance between genes (Canberra, Euclidean, Manhattan and Maximum) and between clusters (Average, Complete, Single and Ward). This was tested using the R packages cluster, Heatplus and stats. Among the 12 hierarchical clusters generated (data not shown), we selected the one that displayed the highest averaged distance across genes.

### RT-PCR

GFAP, Cxcl13 and C4b genes were selected for verifying the microarray technique by RT-PCR, based on the differences observed in their expression between the infected and the control group. DNA amplification from 10 ng of total RNA from animals sacrificed at 120 and 270 dpi was done using commercial primers (QuantiTec Primer Assay) following the QuantiTect SYBR Green RT-PCR kit manufacturer instructions (Qiagen, Hilden, Germany). The amplification was performed on a Smart Cycler thermocycler (Cepheid, Sunnyvale, USA) with the following protocol: 30 min at 50°C, 14 min at 95°C and 45 cycles of 15 s at 94°C, 30 s at 55°C and 30 s at 72°C. Fold-changes were calculated using the 2^-ΔCt ^method [25].

### Immunohistochemical and histochemical analysis

Heat-induced epitope retrieval with citrate buffer (pH 6.0) was applied to the tissue slides. The astrocyte specific rabbit polyclonal antibody against glial fibrillary acidic protein (1:500, Dakocytomation Z0334, GFAP) (Dako, Glostrup, Denmark) and the mouse monoclonal antibody against metallothioneins 1+2 (1:200, Dakocytomation M00639, MT1+2) were used. The antibody binding was visualized with anti-rabbit Dako EnVision Plus System and 3,3'diaminobenzidine as the chromogen substrate. Omission of the primary antibody was used as a negative control.

*Lycopersicum esculentum *agglutinin (1:100, Sigma, L0651) (Sigma, St Louis, USA) histochemistry was also performed on the brain tissue to stain microglial cells. The washing buffer was supplemented with CaCl_2_, MgCl_2 _and MnCl_2 _1 mM. The binding was visualized with the avidin biotin peroxidase (ABC) complex (Pierce, Rockford, USA) and 3,3'diaminobenzidine as the chromogen substrate.

## Results

### Presence of prion protein after inoculation

The presence of the pathological isoform of the prion protein (PrPres) was confirmed by western blot in the brain of all animals inoculated with infectious homogenate (BSE1) sacrificed at 270 dpi. PrPres protein was not detected in the inoculated animals sacrificed at 1, 60 and 120 dpi. PrPres was not detected in any of the control (mock -inoculated) animals at any sacrifice time points.

### Microarray analysis

**Figure 1:**
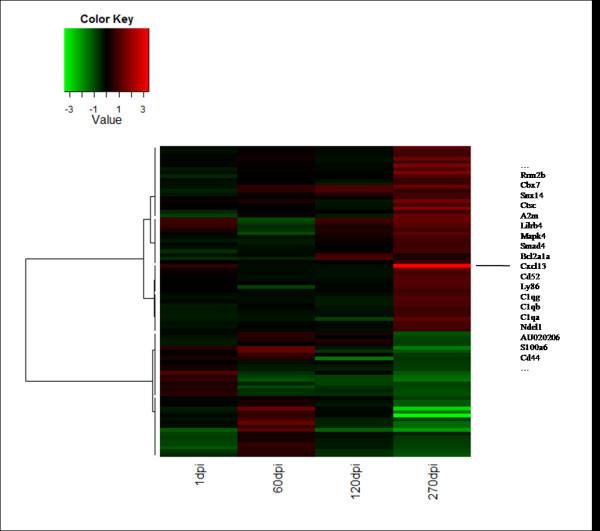
**Hierarchical cluster of differentially expressed genes across time points**. This is a graphical representation of the ratio between animals inoculated and non-inoculated for those 87 genes differentially expressed with a fold-change higher than 1.7 or lower than 0.6 in at least one time point. Among the methods tested, we selected the "Manhattan" method to measure the distance between genes and the "Ward" algorithm to cluster genes. Rows are genes and columns time points. At the top, the color log2 scale indicates the expression level of treated animals compared with the control ones. Genes colored in red are more expressed in BSE inoculated animals than in control ones, whereas genes colored in green are more expressed in control animals. Genes that are more up regulated are highlighted in the figure.

Microarray data was obtained from animals sacrificed after 1, 60, 120 and/or 270 dpi. Virtually no changes in the gene expression were observed at 1 dpi. However, major gene expression changes were observed from 60 dpi onwards as shown in the hierarchical cluster (see Figure 1). Those changes were related to different biological processes such as neuronal metabolism, inflammatory response and signal transduction, among others. Table 1 summarizes the fold-change in the expression (either down regulated or up regulated) of the 87 genes that had statistically significant expression changes at least at one time point. The genes are listed according to their biological functions [8-10,12,15,18,19,26-38].

Changes in two main biological processes can be highlighted in the group of animals inoculated with BSE homogenate: neural cell metabolism and defense mechanisms. In the early and intermediate phases of prion infection, prior to PrPres detection in the nervous tissue (60 and 120 dpi), the gene expression pattern resulting from the inoculation of the prion protein shows a mild but evident alteration of the normal neuronal and glial metabolism, neuronal plasticity and signal transduction, which are processes that can influence neuronal viability. Examples are the downregulation of Npas3 (a transcription factor involved in the neuronal signaling [39]) and Rrm2b (a gene related to DNA replication and reparation, whose absence results in apoptotic cell death [40]). Those patterns taken into consideration together with other gene expression changes like the sustained down-regulation, from the day 60 after inoculation onwards of inducible transcription factors like Fos and Jun-B (a group of neuronal apoptosis inducers [41-43]), could be depicting a search for a balance between neuronal survival and neuronal degeneration.

**Figure 2:**
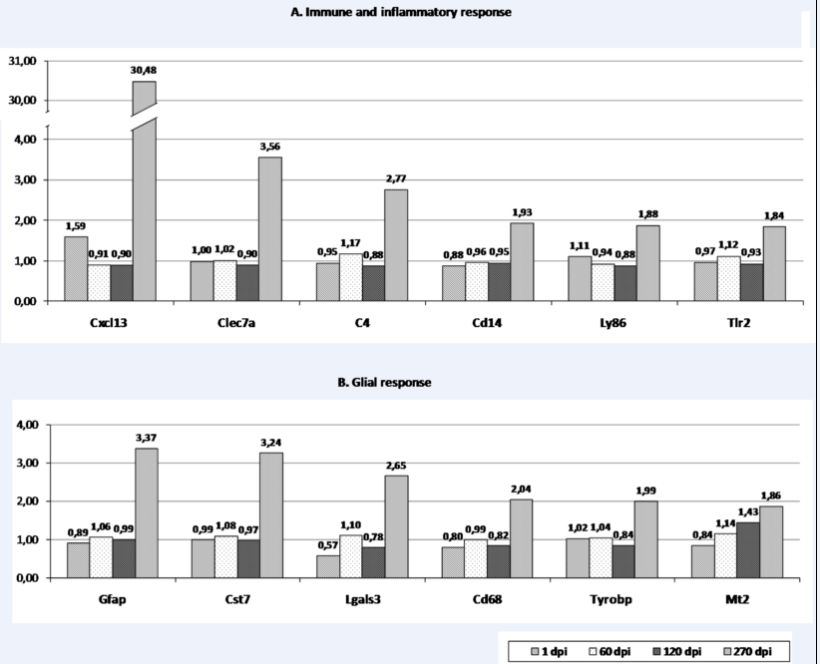
**Graphical representation of the expression of genes related to immune and inflammatory response (A) and glial response (B)**. Fold change values at 1, 60, 120 and 270 dpi (see pattern legend for time points).

The number of genes that varied their expression patterns was higher in the late phases of the disease (270 dpi, see Table 1). At this moment, a manifest activation of gene expression was observed in those genes related to the Central Nervous System defense mechanisms such as glial activation and neuroinflammatory response (see Figure 2), which presumably led to neurodegeneration and cell death.

Some of the genes involved in immune and inflammatory pathways identified in the present study are complement activation factors (C1qa, C1qb, C1qg, C3, C4, C3ar1), chemotactic molecules (CXCL13, Lgals3, C3ar1), neuroinflammation markers (GFAP, Clec7a, Lgals3), genes codifying for receptors involved in innate immune response (CD14, TLR2), inflammatory cell types (CD44, CD68, Ly86) and genes that can be related to microglial activation (Tyrobp, HSPs, TLR2, Lgals3, OSMR, Map4k2) and astrocyte activation (GFAP, HSPs, OSMR...). The release of reactive oxygen species (ROS) resulting from the aforementioned neuroinflammatory condition, might have contributed to an over expression of metallothioneins. Cell death and cell survival related mechanisms were also activated in the BSE inoculated group (Ctsc, CD68, Rtn3, Tyrobp, Tmem109, Egr2, Fos and Jun-B).

Another group of genes with altered expression levels are those involved in cellular trafficking (Pmch, Tyrobp, Rtn3, Ndel1, Snx6, Snx14, Arc) (see Table 1) and this might have a role in intracellular and axonal transport and even synaptic impairment.

### Validation of microarray results

Specific gene expression data was further validated by a combination of RT-PCR and immunohistochemical and histochemical techniques. For RT-PCR experiments GFAP, Cxcl13 and C4b were selected and RNA obtained at 120 and 270 dpi were examined. As shown in Table 2 RT-PCR data are, in most cases, in reasonably good agreement with microarray data.

**Table 2:** Quantification by RT-PCR of selected genes.

	Fold-change Cxcl13		Fold-change GFAP		Fold-change C4b	
Time point (dpi)	RT-PCR	microarray	RT-PCR	microarray	RT-PCR	microarray
120	1.95	0.90	3.54	0.99	1.28	0.88
270	51.92	30.48	3.59	3.37	2.13	2.77

Fold-change expression values of GFAP, Cxcl13 and C4b genes at 120 and 270 dpi comparing control and BSE inoculated groups. Data are mean from five inoculated vs. control animals at 120 dpi and from six inoculated *vs *control animals at 270 dpi.

**Figure 3:**
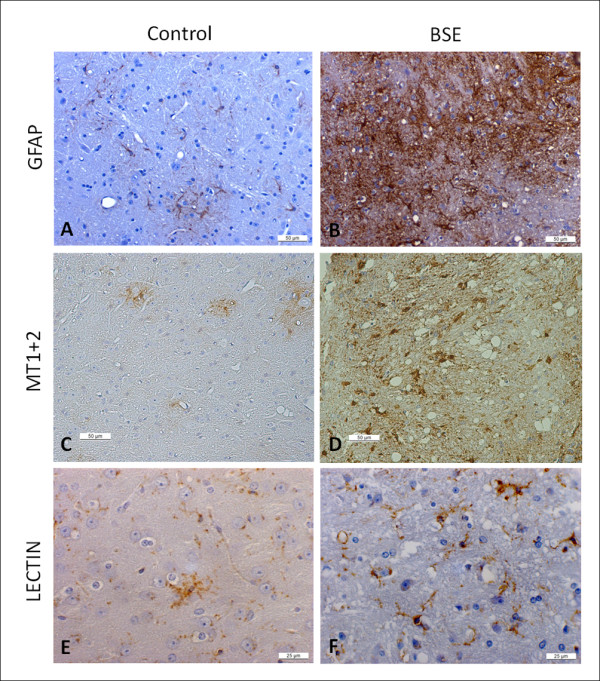
**Brain sections of boTg110 terminal stage mice (275 dpi) and mock inoculated matched controls**. Upper panel: GFAP immunostaining in the medulla oblongata. Note the astrocyte activation in the BSE-inoculated mice at terminal stages (B) in comparison with the control group (A). Middle panel: MT1+2 immunostaining in the cerebellum. Note the increased presence of stellate shaped cells in the BSE-inoculated group (D) at a terminal stage. Lower panel: Lectin staining in the medulla oblongata. Note the proliferation of microglia in BSE-inoculated mice (F).

To further validate at the protein level the observed changes in expression, the GFAP and MT proteins were examined in situ by immunohistochemistry on formalin-fixed paraffin embedded brain tissue using antibodies against GFAP and MT1+2. Using GFAP antibody, animals inoculated with BSE homogenate and culled at advanced stages of the disease (250-350 dpi) showed an increased immunolabeling of stellate shaped glial cells (astrocytes) which were increased in number and hypertrophic when compared to controls. This increase was particularly intense in the medulla oblongata (see Figure 3) as well as in the thalamus, mesencephalon and deep cerebellar nuclei (data not shown).

Immunostaining with MT1+2 antibody revealed an increased labeling in terminal stage BSE- inoculated animals, particularly in the medulla oblongata region, when compared to the control mice (see Figure 3).

In order to visualize the microglial cells and corroborate the microglial activation supported by the microarray results, tomato lectin histochemistry was performed on paraffin embedded brain tissue. Histochemistry revealed proliferation of microglial cells in different areas of the brain, (particularly in the grey matter of the medulla oblongata (see Figure 3), thalamus, mesencephalon, and deep cerebellar nuclei) of mice inoculated with BSE homogenate from the 150 dpi group onwards.

## Discussion

Changes in gene expression in the brain were detected between the BSE inoculated group and the control group throughout all the time points after inoculation.

### Early and intermediate changes

A mild alteration of the gene expression was detected at 1 dpi in the group dosed with infectious homogenate in comparison with the control group (see Table 1), probably related to the introduction of molecules such as cytokines, free radicals, peroxides, etc, present in the infectious homogenate. The pathological prion protein, whose neurotoxic properties have been demonstrated in vitro [44-46], could also have a role in the observed early alteration.

The alteration of neuronal and microglial activity is evident in BSE infected animals beginning at the early stages of the disease (see Table 1). The observed expression pattern in the early stages (see Table 1) could be indicating a search for a balance between the mechanisms leading to cell death and the survival efforts of the neuronal populations. A hypothetical example is the possible effect of the observed expression pattern of genes like Rrmb2, Npas3, Ptprd, Mapk4, Fos, and Jun-B over the hippocampus. Since Rrm2b is involved in DNA repair [40], its downregulation at 60dpi may result in an increased cell death. Furthermore, the downregulation of Npas3 may block an essential route for the hippocampal neurogenesis by its role in neuronal signaling [39]. The effect of both genes taken together would result in a fatal outcome in the hippocampal region. On the contrary, the upregulation of Ptprd [47] at 60 dpi and Mapk4 gene [48] at 120 dpi, together with the downregulation of inducible transcription factors like Fos and Jun-B [41-43] could be understood as a compensation process against the damage caused by the inoculated agent by attempting to avoid the apoptosis mechanisms.

### Late changes

Changes in physiological processes like signal transduction, metabolism, cell transport and the neuroinflammatory response, as a consequence of the alterations caused by the PrPres inoculation have been described previously in TSE [5,10,13,18,28,30,49-51]. Our results provide additional evidence of expression changes in genes included in functional categories such as synaptic functionality, neuroinflammation and cell death, among others at later stages of the disease (270 dpi) (see Table 1).

Neuroinflammation is the most evident process at the later stages of the disease. Induction of C1 subunits (C1qa, C1qb and C1qg, from the classical pathway of complement activation), C4 (classical and lectin pathways) and C3 factor (a common factor in the three complement activation paths) suggest that the classical complement activation pathway has an important role in the CNS pathogenesis of BSE. Complement activation has been described previously in prion diseases like scrapie [18], Creutzfeldt-Jakob disease [30] and also in BSE inoculated mice [52] as an indicator of the innate immune response.

The upregulation of genes coding for receptors involved in innate response (CD14, TLR2) similar to what has been described for scrapie models at terminal stages [18] is an interesting issue since these molecules and its cofactor Ly86 (also up regulated) have been associated to the innate response against other pathogens [53-58]. The role of TLR in TSE pathogenesis has been previously questioned [59] yet its up-regulation in the present model could be related to PrPres deposition. On the contrary, researchers questioning the "protein only" hypothesis suggest that classical infectious agents such as viruses [51,60-63] or bacteria [64,65] could be involved in TSE pathogenesis, in which case an innate response, such as the one suggested by the present results would also certainly fit.

As previously shown in the same model [66,67] and in a wild type murine model [10] cellular and oxidative stress seem to play a significant role in the outcome of BSE. Additional evidence of this is provided by the results of the present experiment, namely by an upregulation of HSPB6 (HSP20) and Mt2 at 270 dpi (see Table 1). The over expression of Mt2 in the BoTg110 transgenic mouse model is in accordance with the BSE gene expression analysis performed by Sawiris and coworkers on wild type mice [10] and other TSE studies [5,27,28,49,68] confirming glial activation as one of the key processes taking place in these diseases.

Neuronal degeneration and neuronal death are characteristic processes of prionic diseases [69]. Lysosomal activity has been pointed out as one of the first steps in neurodegeneration [19,70] and lysosomal liberation to the extracellular space has been described in many neurodegenerative diseases [18,71-73]. The gene expression analysis of our mice model of BSE reflects an increase in lysosomal activity at 270 dpi, as Ctsc and CD68 are over expressed (see Table 1). If Ctsc were over expressed in neurons this could be related to the programmed cell death type since lisosomal proteases are capable of activating cell death programs [18,74-77]. CD68 induction, indicating microglial activation [78], has been previously described in a scrapie mice model and in sporadic CJD natural cases [18,30].

The upregulation of Cst7 in the present BSE model, described in other TSE [18,19,61], can be a consequence of the induction of lysosomal proteases [18] or could have a compensatory role against the accumulation of abnormal protein in some neurodegenerative diseases [8,18,79,80]. The downregulation of the Rtn3 gene observed at 270 dpi may lead to a decrease in the Bcl-2 antiapoptotic function, leading to neuronal death. Its inhibition could also affect neuronal plasticity and functionality, since the axonal transport would be affected. Despite the evidence of neuronal degeneration and death at later stages of the disease, the neuroprotective effect of Scn2b downregulation and the antiapoptotic effect of Tmem109 and Egr2 inhibition at 270 dpi, among other changes in the expression pattern, could be understood as unsuccessful neuronal survival efforts.

Synaptic functionality and cellular trafficking are also affected cell functions. The downregulation of Pmch, Tyrobp and Arc genes and the upregulation of the Ndel1 gene in the BSE inoculated mice supports that synaptic impairment is part of the BSE pathogenic process, since these genes are related to synaptic plasticity and functionality [81,82].

Upregulation of sortin nexins (Snx6, Snx14) (see Table 1) could affect the normal intracellular trafficking of receptors [83] since they are involved in endocytosis processes and vesicular transport of membrane compounds. These results were in agreement with previous studies suggesting alterations of the synaptic machinery and the neuronal protein transport in advanced stages of the prionic diseases [30,50,84].

In summary, we present a gene expression analysis on BSE using a transgenic mouse model. The results obtained show a considerable parallelism with the results obtained in previous studies on animal and human TSE. The observed changes in gene expression are strongly indicative of a neuroinflammatory reaction occurring in the brain in advanced stages of the disease with an important participation of inflammatory cells, resident macrophages (microglia) and activated astroglia. Our results also point out an alteration of neuronal metabolism and functionality previous to the inflammation, which remains present until the later stages of the disease. Processes like neuronal degeneration and cell survival mechanisms were activated. From the earlier stages of the disease throughout the entire infection period, changes in the expression of genes involved in the neuronal metabolism show the search for balance between neurodegeneration and cell survival.

The results of the present study establish a base for further specific investigations of the different mechanisms involved in the BSE pathogenesis. Particularly important genes are those associated for the first time to the course of prion diseases and the early changes detected previous to the onset of neuroinflammation, which require further investigations in order to explain the mechanisms involved in the PrPres accumulation. These are also interesting therapeutic targets and potential disease markers to be considered in preclinical diagnostic tool development. Further investigations are needed in order to assign the appropiate biological relevance in the course of the prion diseases to those genes associated for the first time to prion diseases. It is evident that the neuroinflammation phenomenon is a pillar of BSE pathogenesis and that the therapeutic approach towards its prevention could be a way of stopping the neurodegeneration process. The results presented are also important for the characterization of the boTg110 transgenic model, a murine model for BSE which is nowadays being used in other experiments.

Interpretation of the microarray data is subjective to statistical selection criteria and to the criterion of the investigator and for this reason, genes discarded for not entering the established acceptation limits should not be excluded from further investigations about TSE pathogenesis. Another issue that needs to be considered when interpreting the results is that, obviously, post transcriptional regulatory mechanisms might modify the biological effects of the expressed genes and therefore their biological impact.

## Competing interests

The authors declare that they have no competing interests.

## Authors' contributions

RT carried out the molecular genetic studies and the immunohistochemical assay and drafted the manuscript, XC participated in the sample preparation for the molecular genetic studies and performed the statistical analysis of the microarray data, EV and CC participated in the immunohistochemical analysis and carried out the histochemical analysis, EV helped to draft the manuscript, MRV and AS carried out the verification, normalization and the preliminary statistical analysis of microarray data, AB participated in the molecular genetic analysis and helped draft the manuscript, JMT participated in the design of the study and provided the trangenic murine model, MP and JA conceived the study, and participated in its design and coordination and helped to draft the manuscript. All authors read and approved the final manuscript.

## Supplementary Material

Additional file 1 **Selected probesets**. List of differentially expressed probesets between the BSE inoculated mice group and control mice group for each timepoint (1, 60, 120 and 270 dpi) and their associated *p*-values adjusted by the Benjamini and Hochberg method. Click here for file

Additional file 2 **Selected genes**. List of differentially expressed genes between the BSE inoculated mice group and control mice group for each timepoint (1, 60, 120 and 270 dpi) and their associated *p*-values adjusted by the Benjamini and Hochberg method. Click here for file
